# Prognostic and diagnostic potential of isocitrate dehydrogenase 1 in esophageal squamous cell carcinoma

**DOI:** 10.18632/oncotarget.13351

**Published:** 2016-11-15

**Authors:** Xuan Chen, Qingbao Li, Cong Wang, Wenzhe Xu, Lihui Han, Yuan Liu, Bowen Liu, Shanghui Guan, Bingxu Tan, Jianbo Wang, Nana Wang, Qingxu Song, Yibin Jia, Jianzhen Wang, Linli Zhao, Yufeng Cheng

**Affiliations:** ^1^ Department of Radiation Oncology, Qilu Hospital of Shandong University, Jinan, Shandong, 250012, China; ^2^ Department of Cardiac Surgery, Shandong Provincial Hospital Affiliated to Shandong University, Jinan, Shandong, 250021, China; ^3^ Department of Neurosurgery, Qilu Hospital of Shandong University, Jinan, Shandong, 250012, China

**Keywords:** ESCC, IDH1, protein expression, diagnosis, prognosis

## Abstract

We aimed to investigate the pattern of expression and clinical significance of isocitrate dehydrogenase 1(IDH1) in esophageal squamous cell carcinoma (ESCC). The IDH1 expression was determined by quantitative real-time polymerase chain reaction, immunohistochemistry, and Western blot analysis using 38 pairs of frozen tissues. Enzyme-linked immunosorbent assay was employed to measure 67 pairs of serum samples from patients and their controls to evaluate its diagnostic value. Immunohistochemistry analysis of 111 formalin-fixed paraffin embedded tissue samples was conducted for explaining its prognostic value. After shRNA transfection, CCK8 and clonal efficiency assays were carried on for verifying the function of IDH1 *in vitro*. Increased expression at mRNA (*P* < 0.001) and protein levels (immunohistochemistry: *P* < 0.001, Western blot analysis: *P* < 0.001) were observed. Similarly, the IDH1 expression in serum from patients with ESCC was significantly upregulated relative to that from healthy controls (*P* < 0.001). Kaplan–Meier curve indicated that IDH1 upregulation predicted worse overall survival (OS) and progression-free survival (PFS). Univariate and multivariate analyses identified IDH1 expression as an independent prognostic factor for OS and PFS. Furthermore, OD450 values and colony numbers were decreased in sh-IDH1 groups (all *P* < 0.05). In conclusion, IDH1 is upregulated in patients with ESCC and can be used as a good potential biomarker for diagnosis and prognosis.

## INTRODUCTION

Esophageal cancer is the sixth main cause of cancer-related mortality and the eighth most common cancer worldwide [1]. The high-risk geographic region, referred to as the “esophageal cancer belt,” extends from Northern Iran to North Central China through Central Asia. In this region, more than 90% of the cases are identified as esophageal squamous cell carcinoma (ESCC), in contrast to only 26% in the United States [3]. Advanced techniques in the diagnosis and treatment of esophageal cancer failed to improve the prognosis. The 5-year overall survival rate of patients with ESCC ranges from 15% to 25% [4]. Biological factors are superior to endoscopy in assessing the malignant behavior of ESCC in terms of invasiveness, cost, and testability. A number of tumor-specific proteins have been identified as tumor markers for various cancers. These proteins include cancer antigen 125 (CA125) in ovarian cancer, alpha-fetoprotein (AFP) in liver cancer, carcinoembryonic antigen (CEA) in colon cancer, and prostate-specific antigen (PSA) in prostate cancer. No superior biomarkers have thus far been found in ESCC patients. Several proteins have been identified as key molecules in signal transduction pathways of ESCC development, such as vascular endothelial growth factor (VEGF)-C, peptide antigen, CEA. However, insufficient sensitivity and specificity parameters limit the application of these biomarkers in the early diagnosis of ESCC [6]. New biomarkers with combined high sensitivity and good specificity for the diagnosis and prognosis in ESCC can provide enhanced clinical benefits.

Metabolic plasticity is regarded as a hallmark of cancer [7]. Several transformational metabolic features are observed quite generally across various types of cancer cells [8]. Cancer cell metabolism is characterized by the ability of acquiring essential nutrients from a frequently innutritious environment and using these nutrients to both maintain the vitality of its own and build greater biomass [9]. The isocitrate dehydrogenase (IDH) family comprise the rate-limiting enzymes in the tricarboxylic acid (TCA) cycle and include 3 members (IDH1, IDH2, and IDH3) that convert isocitrate to α-ketoglutarate(α-KG) by oxidative decarboxylation [10]. α-KG is an intermediate of the TCA cycle and a cosubstrate of a number of dioxygenase reactions in the cytoplasm and the nucleus [11]. IDH also produces non-mitochondrial NADPH. NADPH as a required component to handle redox stress is crucial for tumor cell growth [12]. IDH not only participates in lipid biosynthesis but acts as an essential antioxidant as well [13]. Cancer cells exhibit persistently high levels of reactive oxygen species (ROS) because of genetic, metabolic and microenvironmental alterations including hypoxia, as well as crosstalk between cancer and immune cells [14]. These ROS-induced dysfunctions are compensated by an upregulated antioxidative capability of these cancer cells [15]. IDH, together with a long-lasting endogenous substrate (glutamate), mainly contributes to the regeneration of reduced glutathione (GSH), which is a critically important antioxidative system against oxidative damage and xenobiotic toxicity to cells by providing NADPH [16, 17]. Moreover, the inhibition of antioxidative systems can kill cancer cells synergistically with radiotherapy and chemotherapy both *in vitro* and *in vivo* [18].

IDH1 plays driving roles in the metabolism of glucose, fatty acids, and glutamine as well as the maintenance of cellular redox status; IDH1 is located in the cytoplasm and peroxisomes [19]. Recent studies on IDH1 in cancers have primarily focused on the mutations of the *IDH1* gene. *IDH1* mutations were found in low-grade glioma and secondary glioblastoma, acute myeloid leukemia, chondrosarcoma, intrahepatic cholangiocarcinoma, and melanoma [22–24].

The aforementioned studies on the *IDH1* gene indicate that *IDH1* mutation may significanty affect tumorigenesis and tumor progression. *IDH*-mutated cancers cannot be reduced to homozygosity and retain one *IDH* wild-type allele. Ward et al. suggested and then validated that wild-type *IDH1* promotes cell growth and proliferation [25]. Aberrant protein expression, as the primary functional gene output, complements genome initiatives and is an important phenotypic characteristic of cancer. The association of protein biomarkers with clinical characteristics and outcomes of cancer patients may elucidate the underlying molecular mechanisms of cancer initiation and progression [26]. Studies on wild-type IDH1 protein as a diagnostic and prognostic biomarker remain inadequate. IDH1 protein has been identified as a novel biomarker for the diagnosis of non-small cell lung cancer [27]. A study using genome-wide RNA-Seq indicates that IDH1 expression is higher in ESCC tissues than in normal tissues [28]. However, the protein expression of IDH1 in ESCC and its correlation with 5-year overall survival (OS) rates and progression-free survival (PFS) are undetermined.

In the current study, we compared the expression of IDH1 in the tumor tissue with that in the paracancerous tissue by quantitative real-time PCR (qRT–PCR), immunohistochemistry, and Western blot analysis. The serum expression in patients and healthy controls were used to assess the value of IDH1 as a diagnostic biomarker. Moreover, the association of IDH1 with the clinicopathological characteristics of patients with ESCC and the prognostic value of IDH1 were analyzed. CCK8 and clonal efficiency assays were used for observing if IDH1 could affect growth and proliferation of ESCC cells.

## RESULTS

### IDH1 expression in frozen tissues

**Figure 1:**
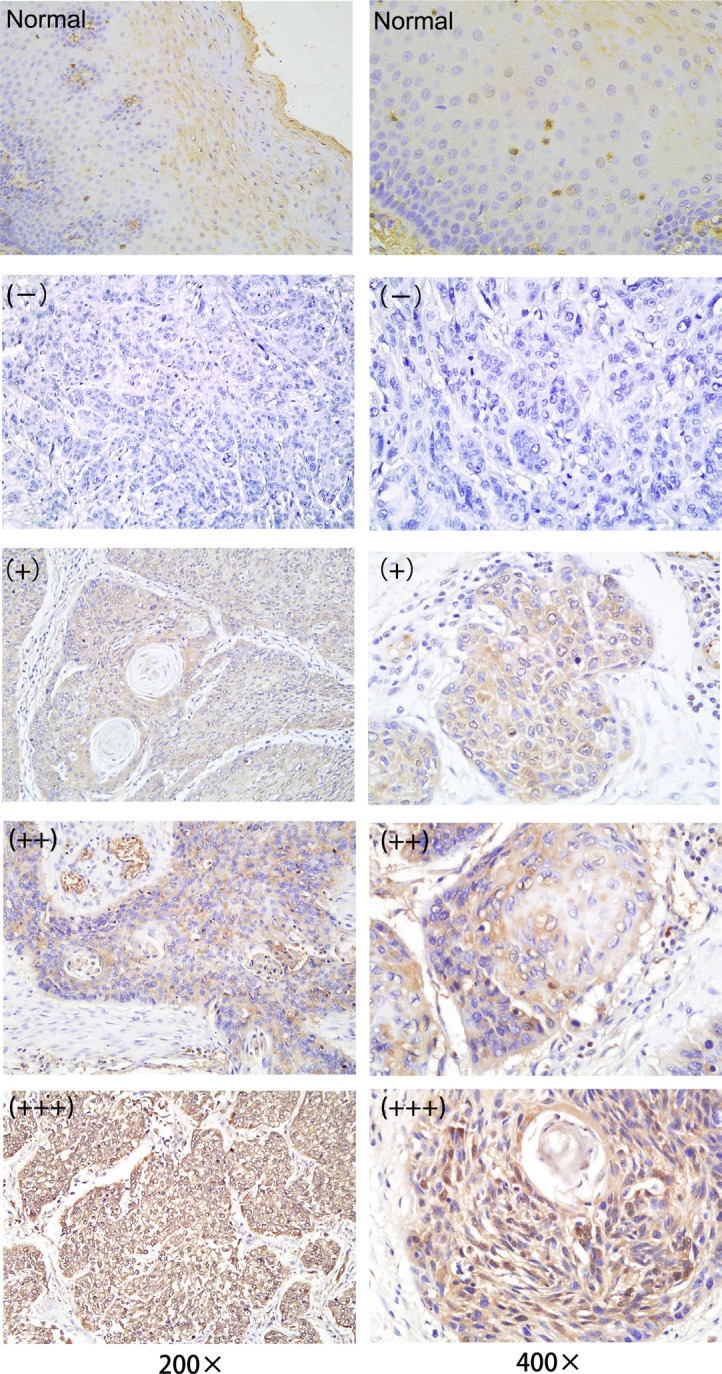
IDH1 expression in patients with ESCC was examined by performing immunohistochemistry Left panel: ×200. Right panel: ×400. From top to bottom, in order, are as follows: paracancerous normal tissues, and (–), (+), (++), (+++) of cancerous tissues.

**Figure 2:**
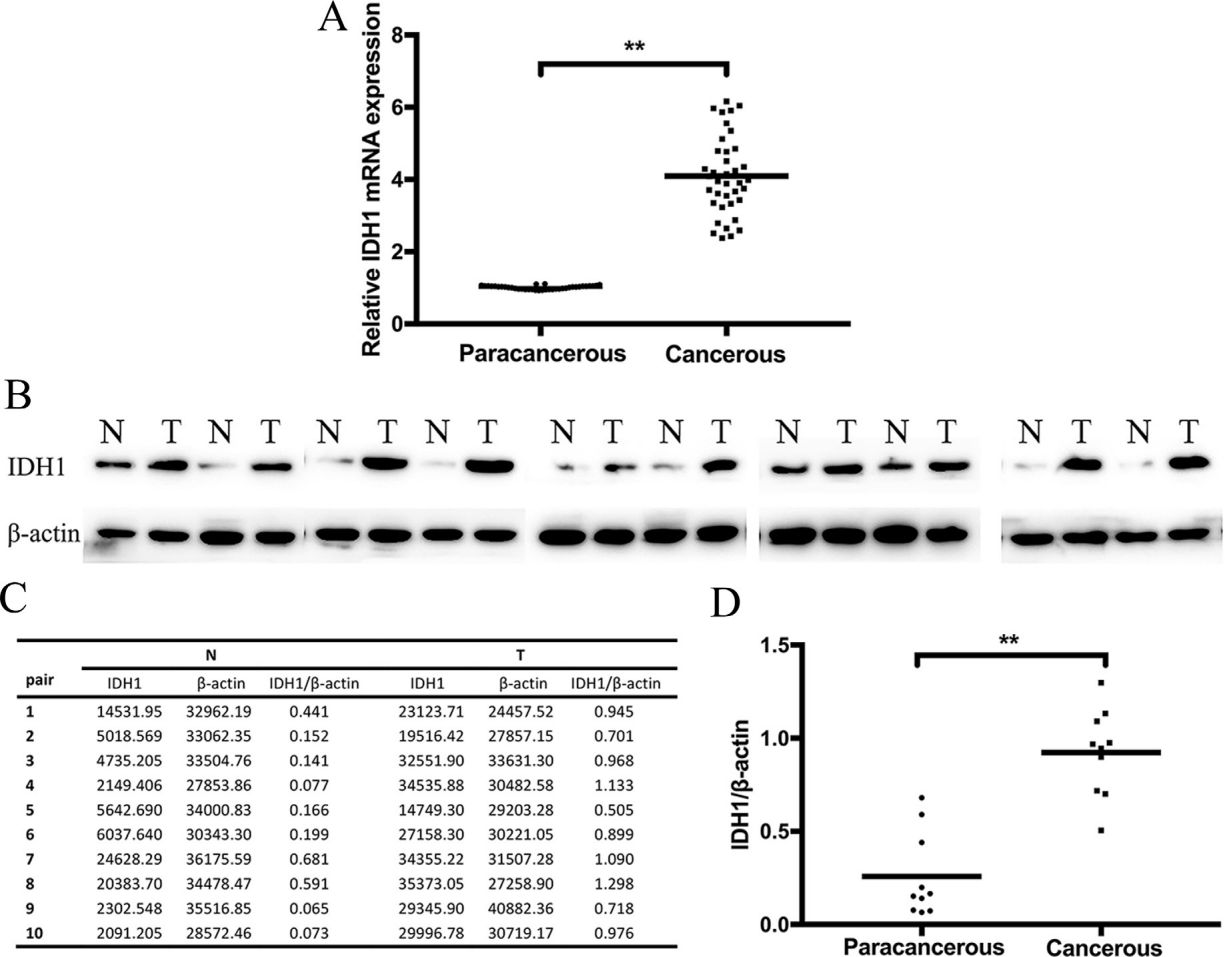
IDH1 expression in cancerous tissue compared with that in paracancerous tissue was detected at (A) mRNA level by RT–PCR The 2−ΔΔCt method was used for calculating the IDH1 expression. The relative expression was 4.04 ± 1.05 vs. 1.01 ± 0.05 (*P <* 0.001). (**B**) Protein level was detected by Western blot analysis, the intensity values of 10 pairs of tissues are shown in (**C**) and the IDH1/β-actin values of cancerous and paracancerous tissues are compared in (**D**). Abbreviations: T, cancerous tissues; N, paracancerous tissues.

IDH1 expression was analyzed by IHC, qRT–PCR, and Western blot analysis. The IDH1 expression in the formalin-fixed paraffin embedded (FFPE) tissue samples was determined by IHC. The IDH1 protein was primarily distributed in the cytoplasm of ESCC cells (Figure 1). Cancerous samples showed 22 (+++), 8 (++), 6 (+), and 2 (–), whereas paracancerous tissues showed 34 (–) and 4 (+). Consequently, it was highly expressed in 22 cancerous tissues and 0 paracancerous tissues, and a significant difference was indicated (Table 1, *P* < 0.001). By qRT–PCR analysis, IDH1 in cancerous tissues was upregulated relative to that in paracancerous tissues in 38 patients (Figure 2A, *P* < 0.001). To verify the IDH1 level, Western blot analysis was performed with 10 pairs of cancerous and paracancerous tissues (Figure 2B). The results suggested that IDH1 expression was higher in cancerous tissues than in paracancerous tissues (Figure 2C, 2D, *P* < 0.001).

**Table 1:** Quantification of the expression of IDH1 in cancerous and paracancerous tissues via IHC staining

Group	*n*	Overexpression (*n*)	Overexpression rate (%)	χ2	*P* value
Cancerous tissue	38	22	57.89%	30.963	< 0.001
Paracancerous tissue	38	0	0%		

Abbreviation: IHC, immunohistochemical.

### Diagnostic value of serum IDH1

**Figure 3:**
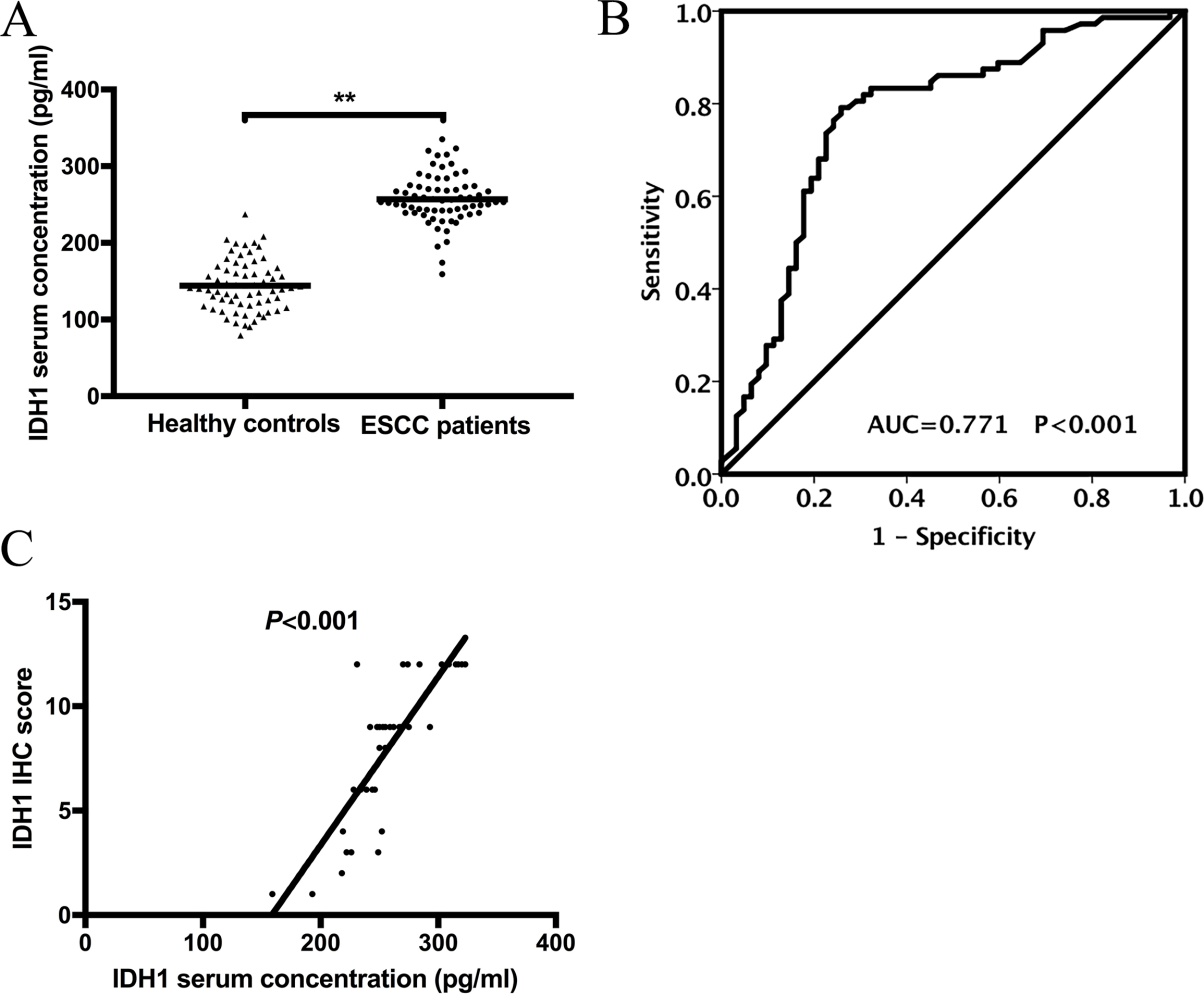
(**A**) IDH1 serum concentration in ESCC patients and healthy controls detected by ELISA. (**B**) ROC–AUC curve analysis for the diagnostic value of IDH1.

We assessed the serum levels of IDH1 in 67 patients with ESCC and 67 healthy controls by enzyme-linked immunosorbent assay (ELISA) (Figure 3A). The mean value of IDH1 serum concentration in ESCC patients and healthy controls was 189.66 pg/mL. IDH1 was significantly upregulated in patients with ESCC (141.6 ± 30.353 pg/mL vs. 257.8 ± 26.609 pg/mL, *P <* 0.001). We also investigated the relationship between the serum level of IDH1 and the clinicopathological features of patients with ESCC. The IDH1 expression was significantly upregulated with advanced TNM staging for ESCC (*P* = 0.048). No significant links to other clinicopathological features were observed, such as age, gender, smoking, drinking, differentiation stage, T stage, and N stage (Table 2). The receiver operating characteristic (ROC) curve shows that the area under the ROC curve (AUC) was 0.771 ± 0.043 (Figure 3B). With the cut-off value set to 192.084 pg/mL (Yuden index), the sensitivity and specificity of serum IDH1 were 83.3% and 67.7%, respectively. Pearson correlation coefficient analysis was used to determine the association of IHC scores in 38 frozen tissues with their paired serum expression levels (Figure 3C); the r value was 0.813 (*P <* 0.001).

**Table 2:** The correlation of clinicopathologic variables of ESCC with serum IDH1 expression

Clinicopathological features	IDH1 overexpression		*P*^a^ value
	No (*n* = 24)	Yes (*n* = 43)	
Age			0.459
< 65	14	27	
≥ 65	10	16	
Gender			0.564
Female	7	12	
Male	17	31	
Smoking			0.365
No	11	23	
Yes	13	20	
Drinking			0.581
No	11	20	
Yes	13	23	
Differentiation			0.922
Well	7	12	
Moderate	10	20	
Poor	7	11	
T stage			0.196
T1	5	3	
T2	7	9	
T3	10	22	
T4	2	9	
N stage			0.094
N0	16	15	
N1	3	11	
N2	4	12	
N3	1	5	
TNM stage			0.048*
I	5	3	
II	12	15	
III	7	25	

*P*^a^: Chi-square test.

Abbreviation: FFPE, formalin-fixed paraffin-embedded.

### Prognostic value of IDH1

**Figure 4:**
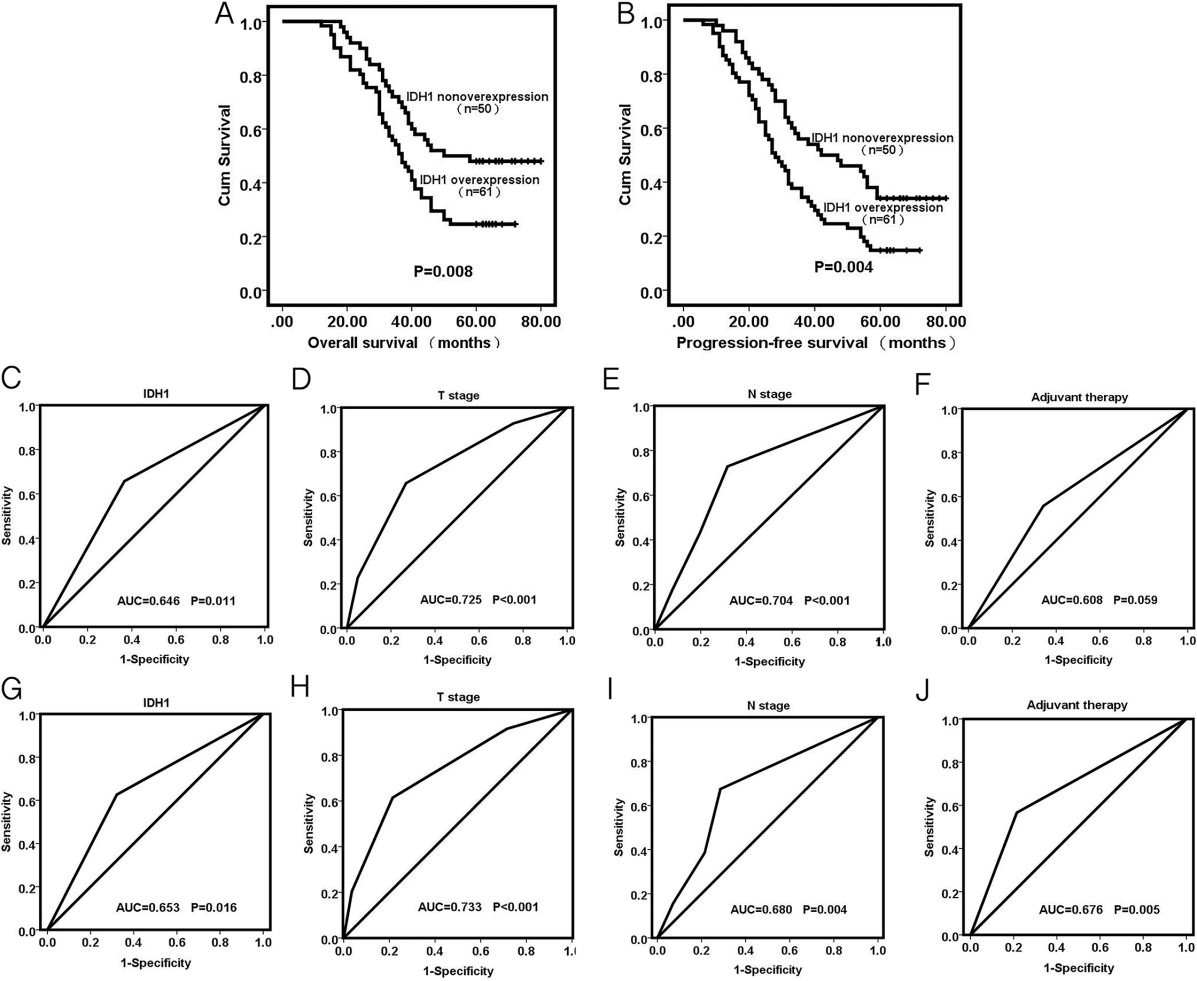
(**A** and **B**) Kaplan–Meier analysis for overall survival and progression-free survival of ESCC patients with IDH1 non-overexpression or overexpression. (**C**–**F**) ROC–AUC curve analysis for OS prediction of IDH1, T stage, N stage and adjuvant therapy. (**G**–**J**) ROC-AUC curve analysis for PFS prediction of IDH1, T stage, N stage and adjuvant therapy.

FFPE tissues from 149 patients were used to assess IDH1 expression. A total of 83 (+++), 36 (++), 22 (+), and 8 (–) were observed in accordance with the evaluation standard. The overexpression rate was 55.7 among all cases. We used the bilateral *χ*^2^ test to determine the correlations between IDH1 and the clinical characteristics of patients (Table 3). The development of differentiation (*P* = 0.038) and T stage (*P* = 0.022) were significantly correlated with IDH1 expression. Age, gender, smoking, drinking, N stage, and TNM stage showed no significant association with IDH1 expression. The FFPE tissues from 111 patients with their prognostic data were used for Kaplan–Meier survival analysis and Cox regression analysis. The Kaplan–Meier curve revealed that the patients with IDH1 upregulation exhibited shorter progression-free survival (PFS) and overall survival (OS) compared with patients without IDH1 upregulation (Figure 4A, 4B). Cox regression analyses were conducted to identify prognostic factors for OS and PFS in ESCC (Table 4). Univariate survival analysis indicated that OS and PFS are negatively correlated with IDH1 expression (*P* = 0.008 and *P* = 0.004). Multivariate analysis confirmed that IDH1 can potentially be an independent prognostic factor for OS and PFS (*P <* 0.001, respectively). T stage, N stage, and adjuvant therapy were also identified as independent predictive factors for OS and PFS in patients with ESCC (*P* = 0.032, *P* =0.003 and *P <* 0.001, respectively for OS; *P* = 0.044, *P* = 0.002, and *P <* 0.001, respectively for PFS). The AUC of IDH1, T stage, N stage, and adjuvant therapy for OS and PFS prediction are shown in Figure 4C–4J.

**Table 3:** The correlation of clinicopathologic variables of ESCC with IDH1 expression in FFPE cancerous tissues

Clinicopathological features	IDH1 overexpression		*P*^a^ value
	No (*n* = 66)	Yes (*n* = 83)	
Age			0.869
< 65	34	45	
≥65	32	38	
Gender			0.741
Female	28	38	
Male	38	45	
Smoking			0.743
No	34	40	
Yes	32	43	
Drinking			0.328
No	28	42	
Yes	38	41	
Differentiation			0.038*
Well	32	26	
Moderate	12	29	
Poor	22	28	
T stage			0.022*
T1	16	8	
T2	20	29	
T3	23	25	
T4	7	21	
N stage			0.581
N0	31	34	
N1	14	21	
N2	10	18	
N3	11	10	
TNM stage			0.183
I	26	21	
II	15	24	
III	25	38	

*P*^a^: Chi-square test.

Abbreviation: FFPE, formalin-fixed paraffin-embedded.

**Table 4:** Univariate and multivariate analyses of prognostic variables

	OS Univariate analysis	OS Multivariate Analysis			PFS Univariate analysis	PFS Multivariate analysis		
Variable	*P* value	*P* value	HR	95%CI	*P* value	*P* value	HR	95%CI
Gender (Famale VS. Male)	0.884	0.870	1.043	0.630–1.727	0.710	0.896	0.970	0.611–1.539
Age (< 65 vs. ≥ 65)	0.854	0.545	0.844	0.486–1.463	0.959	0.658	0.893	0.543–1.471
Smoking (Yes vs. No)	0.672	0.062	1.941	0.967–3.898	0.715	0.164	1.569	0.832–2.959
Drinking (Yes vs. No)	0.483	0.277	1.479	0.730–2.993	0.338	0.096	1.728	0.908–3.289
T stage	0.002*	0.032*			0.001*	0.044*		
T1			1.000	Ref.			1.000	Ref.
T2		0.023*	3.186	1.170–8.675		0.025*	2.759	1.138–6.688
T3		0.001*	5.766	2.119–15.69		0.001*	4.827	1.988–11.72
T4		0.038*	2.417	1.033–6.586		0.045*	2.367	1.109–5.779
N stage	0.001*	0.003*			0.001*	0.002*		
N0			1.000	Ref.			1.000	Ref.
N1		0.004*	2.779	1.391–5.554		0.001*	2.927	1.543–5.552
N2		0.004*	2.951	1.406–6.194		0.012*	2.369	1.211–4.633
N3		<0.001*	6.672	2.833–15.71		<0.001*	5.987	2.690–13.32
Differentiation	0.080	0.059			0.137	0.164		
Well			1.000	Ref.			1.000	Ref.
Moderate		0.006*	2.537	1.307–4.921		0.016*	2.043	1.140–3.663
Poor		0.023*	2.527	1.138–5.610		0.048*	2.041	1.008–4.132
Adjuvant therapy(Yes vs. No)	0.023*	<0.001*	2.697	1.551–4.690	0.009*	<0.001*	2.876	1.714–4.826
IDH1	0.008*	<0.001*	3.256	1.785–5.939	0.004*	<0.001*	3.536	2.036–6.141
(Overexpression VS. nonoverexpression)								

Abbreviations: OS, overall survival; PFS, progression-free survival; CI: confidence interval.

### Reduced growth and proliferation of ESCC cells by IDH1 inhibition with shRNA

**Figure 5:**
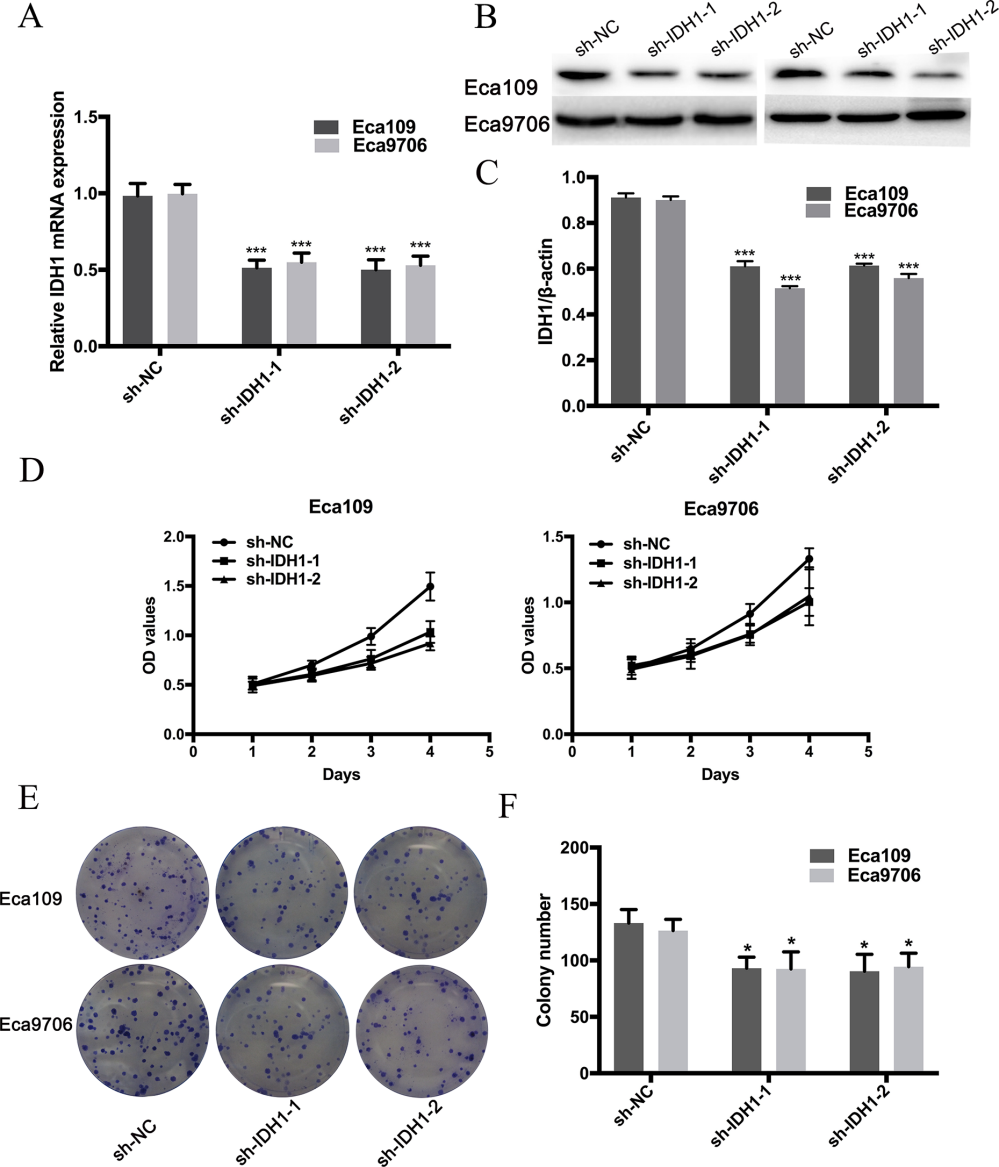
Expression of IDH1 in Eca109 and Eca9706 was reduced by transfection of sh-IDH1-1 and sh-IDH1-2 at mRNA level (A) and protein level (B and C) The OD values (**D**) and clony numbers (**E** and **F**) were decreased in sh-IDH1-1 and sh-IDH1-2 groups.

ESCC cell lines (Eca 109 and Eca 9706) were transfected with shRNA- IDH1. qRT–PCR and Western blot analysis were employed to evaluate the efficiency of transfection. As shown in Figure 5A and 5B, the IDH1 mRNA levels in the sh-IDH1 groups were significantly reduced relative to those in the control group (IDH1/GADPH: 0.51 ± 0.05 and 0.50 ± 0.07 vs. 0.98 ± 0.08 in Eca109, *P <* 0.0001, respectively; 0.55 ± 0.06 and 0.53 ± 0.06 vs. 0.99 ± 0.06 in Eca9706, *P <* 0.0001, respectively.). Figure 4C and 4D show that the IDH1 protein level is also reduced in the transfection group (IDH1/β-actin: 0.61 ± 0.02 and 0.61 ± 0.01 vs. 0.91 ± 0.02 in Eca109, *P <* 0.0001, respectively; 0.51 ± 0.01 and 0.56 ± 0.02 vs. 0.90 ± 0.02 in Eca9706, *P <* 0.0001, respectively). To determine whether knockdown of IDH1 expression by shRNA can decrease the growth and proliferation of ESCC cells, CCK8 and clonal efficiency assays were conducted. The OD450 values of the Eca 109 and Eca 9706 cells transfected with sh-IDH1 indicated significant decreases at 48, 72 and 96h (all *P <* 0.01) relative to those in the control groups (Figure 5C and 5D). Colony numbers of transfected Eca109 and Eca9706 cells were also significantly reduced relative to those of the control groups (all *P <* 0.05, Figure 5E and 5F).

## DISCUSSION

The status of mutant *IDH1* in cancers has been revealed in recent years. Studies on *IDH1* mutations in glioma and acute myeloid leukemia (AML) have been well developed [29, 30]. However, IDH1 is not regarded as a frequent mutation in patients with ESCC according to 3 whole genome sequencing or whole exome sequencing analyses conducted among a Chinese population, as well as a study conducted among a Japanese population [31–34]. Consequently, the current study focused only on the expression of wild-type IDH1 in ESCC.

In the present study, IDH1 expression is higher in cancerous tissues than in paracancerous tissues (mRNA: *P <* 0.001; IHC: *P <* 0.001; Western blot analysis: *P <* 0.001). Despite the individual difference in IDH1 expression in different patients, IDH1 was more highly expressed in cancerous tissues than in paracancerous tissues for one patient. This finding indicates that IDH1 can potentially be a good biomarker for ESCC.

Previous study has confirmed the diagnostic value of IDH1 for NSCLC [27].

We found that IDH1 could act as a potential diagnostic biomarker for ESCC. The result indicated higher IDH1 expression in patients with ESCC than in healthy controls. The AUC value was determined to be 0.771 ± 0.043. The Pearson correlation coefficient indicated that the IDH1 expression in frozen cancerous tissue was significantly associated with serum expression. Consequently, serum IDH1 could be a non-invasive diagnostic biomarker for ESCC. Notably, IDH1 expression in serum was positively associated with TNM stage (*P* = 0.048); this link suggests that IDH1 can act as an oncogene in ESCC development. We hypothesized that elevated IDH1 expression in serum can be illustrated in the following aspects. First, cancer cells upregulate cellular IDH1 in response to rapid proliferation and increased ROS. Second, vigorous metabolism and tissue necrosis enhance the permeability of the cytomembrane, leading to the release of IDH1 into circulation. Third, the degradative pathway and efficiency of IDH1 may be transformed in cancer cells at odds with normal cells.

In this study, we explored the prognostic value of IDH1 in ESCC. The upregulation of IDHI was related to the reduction in OS and PFS, as shown by the Kaplan–Meier curve. Further univariate and multivariate analyses indicated that IDH1 was an independent prognostic factor for OS and PFS. The AUC values of 0.646 (*P* = 0.011) and 0.653 (*P* = 0.016) suggested that IDH1 yielded a significant prognostic value with high sensitivity and specificity. Likewise, N stage and adjuvant therapy were relevant to the prognosis of ESCC. Correlations between IDH1 expression and clinicopathological features were analyzed. Unlike the outcome from the serum samples, the expression levels were associated with differentiation and T stage, which may be attributed to discrepant and insufficient sampling. Similarly, this outcome demonstrates the potential of IDH1 as an oncogene in ESCC. The results of CCK8 and clonal efficiency assays showed that ESCC cells with decreased IDH1 possessed inhibited growth and proliferation.

The mechanisms of IDH1 regulation and effects on cancer have not been elaborated. RNA interference in IDH1 inhibits the proliferation of NSCLC cells and suppresses tumor growth in a xenograft model [35]. Moreover, siRNA knockdown of IDH1 significantly decreases the proliferative ability of the AML cell line with wild-type IDH1 [25]. IDH significantly affects antioxidation by supplying NADPH, which is essential for the production of GSH reductase and regeneration of thioredoxin (Trx) [36]. GSH and Trx are important antioxidative systems protecting the cell from oxidative damage by scavenging the ROS and influencing cell survival [37, 38]. New evidence has demonstrated that main cellular antioxidant systems, such as the GSH and Trx systems, promote the growth of cancer cells and suppress the immune response [18]. Trx inhibits cell apoptosis signaling both by scavenging intracellular ROS in cooperation with the GSH system and interfering with the normal functions of apoptosis signal-regulating kinase 1 (ASK1) and p38 mitogen-activated protein kinase [39]. IDH also regulates apoptosis induced by tumor necrosis factor-α and chemotherapy in Hela cells, facilitating the development of a sensitizer to anticancer drugs [40].

Studies with large samples and are warranted to validate our findings, and further studies can be conducted to detect the detailed mechanisms of IDH1 in ESCC. With an enhanced understanding of IDH1 in ESCC, we can elucidate the biological behaviors of ESCC and use such information. In addition, the harmful effects of radiotherapy and several anticancer drugs on the DNA of cancer cells are realized by ROS. IDH1 may be a good predictor for chemotherapy or radiotherapy.

Therefore, IDH1 is significantly upregulated in ESCC tissue and serum. IDH1 shows potential as a biomarker for both diagnosis and prognosis of ESCC.

## MATERIALS AND METHODS

### Specimen collection and patient enrollment

To evaluate the relationship of IDH1 expression with the status of diagnosis, 38 ESCC cancer tissue samples from tissue blocks and 67 serum samples from patients with ESCC were obtained from the Qilu Hospital of Shandong University from February 2015 to June 2015. Tissue samples were collected from patients who provided serum samples. For control groups, adjacent noncancerous tissues from the aforementioned 38 patients and serum from 67 healthy volunteers were collected. In addition, 111 FFPE cancer tissue samples from patients who underwent subtotal esophagectomy and esophagogastric anastomosis plus regional lymph node dissection in 2009 were used for clinical/pathological factors and survival analysis.All specimens were pathologically confirmed, and none of the patients received neoadjuvant therapy (chemotherapy and/or radiotherapy). A total of 40 (36.04%) patients were alive, whereas 71 (63.96%) patients died during follow-up. The median survival time was 40 months (12–80 months). Data on patient demographics,smoking and drinking history, histologic grade, tumor stage, grade of differentiation, and number of dissected lymph nodes were collected. Tumor stages were assessed according to the Seventh Edition of the Cancer Staging Manual of the American Joint Committee on Cancer. This study was evaluated and approved by the Ethics Committee of Qilu Hospital of Shandong University. A written consent was obtained from each patient.

### IHC

The esophageal cancer tissues were fixed in 10% formalin overnight and then embedded in paraffin following the standard procedure. Deparaffinization using xylene and rehydration was conducted. Antigen retrieval was performed using a citrate–EDTA buffer (2 mM EDTA, 10 mM citric acid, 0.05% Tween 20, pH 6.2). To deactivate intrinsic peroxidase, sections were incubated with hydrogen peroxide. We then applied diluted anti-IDH1 antibody (1:60; Proteintech, China) to the sections at 4°C overnight. Samples incubated with PBS instead of a primary antibody were used as negative controls. After a biotin-labeled secondary antibody was added, the sections were reacted with horseradish peroxidase (HRP)-labeled streptavidin. The sections were stained with 3,3′-diaminobenzidine or DAB, followed by counterstaining with hematoxylin. They were finally visualized under a microscope.

Five ×400 fields were randomly selected, and 2 pathologists independently performed the evaluation. Scores were determined by the intensity of the dye color and the quantity of positive cells. The intensity of the dye color was classified as weak, moderate, and intense (denoted by 1, 2, and 3, respectively). Grading of the number of positive cells was based on the following: 0 (< 5%), 1 (5%–25%), 2 (25%–50%), 3 (51%–75%), and 4(> 75%). The percentage of positive cells and the staining intensity of each sample were multiplied to obtain the weighted scores. The 2 grades were added, and the sections were assigned to one of the following levels: 0–1 score (–), 2–4 scores (+), 5–8 scores (++), and 9–12 scores (+++). The overexpressed value was denoted by (+++).

### qRT–PCR

Total RNA was extracted from fresh tissues or cells by using TRIzol Reagent (Invitrogen, USA) in accordance with the manufacturer's instructions. The IDH1 expression of each sample was assessed using the Bio-Rad Single Color Real-Time PCR system (Bio-Rad, USA) with SYBR Green Real Time PCR Master Mix (TOYOBO, Osaka, Japan). The synthesized primer sequences (Sangon Biotech, China) were as follows: IDH1:5′-GTCGTCATGCTTATGGGGAT-3′ (forward primer), 5′-CAACACCACCACCTTCTTCA-3′ (reverse primer); GAPDH: 5′-GAAGGTCGGAGTCAACGGAT-3′ (forward primer), 5′-CCTGGAAGATGGTGATGGGAT-3′ (reverse primer). The IDH1 expression was calculated using the 2−ΔΔCt method, where ΔCt = Ct_IDH1_–Ct_GADPH_ and ΔΔCt = ΔCt_test_ –ΔCt_control_. All assays were performed in triplicate. The data are presented as the mean ± SD.

### Western blot analysis

Radioimmunoprecipitation assay (RIPA) buffer (50 mM Tris, 150 mM NaCl, 1% Triton X-100, 1% sodium deoxycholate, 0.1% SDS, sodium orthovanadate, sodium fluoride, EDTA, leupeptin) and phenylmethylsulfonyl fluoride (PMSF, Beyotime,China) were used to extract protein from tissue homogenate or cells. Every 100 mg of tissue required 1 ml of RIPA buffer and 10 μL of PMSF. The supernatant was stored at −20°C for later use. Extracts containing an identical amount of protein were separated by electrophoresis on 10% polyacrylamide gels. Proteins were transferred to a nitrocellulose membrane and blocked in 5% defatted dry milk. The membrane was incubated with the primary IDH1 antibody and β-actin antibody (1:100, Proteintech, China) and then with HRP-conjugated secondary antibodies. The bands of antigen-antibody complexes were detected using a chemiluminescence detection system (EMD Millipore, USA).

### ELISA

Serum samples were separated from blood and stored at −80°C. The protein concentrations of IDH1 were measured using a commercially available ELISA kit (Proteintech, China). Approximately 100 μL of each sample was added to a 96-well plate coated with the IDH1 antibody for a 2 h reaction. Subsequently, 100 μL of HRP-conjugated antibody was added and then incubated for 1 h. A substrate solution was added, and the absorbance of each of the samples at 450 and 630 nm was detected using the xMark Microplate Spectrophotometer (Bio-Rad Laboratories, Inc., USA). The assay was performed in triplicate, and the average values were used for statistical analysis.

### Cell culture and transfection

Human ESCC cell lines Eca109 (CCTCC) and Eca9706 (ATCC) were cultured in RPMI 1640 (Gibco, USA) supplemented with 10% fetal bovine serum and incubated in a 5% CO_2_ atmosphere at 37°C. Plasmid-mediated shRNA was used for RNA interference. pGPU6/GFP/Neoplasmid was provided by Shanghai GenePharma Biologic Co. Ltd., China: sh-IDH1-1 (Target sequence: 5′-TAACTTTGAAGAAGGTGGTGG-3′), sh-IDH1-2 (Target sequence: 5′-GGTATGAGCATAGGCTCAT CG-3′), sh-NC (Target sequence: 5′-GTTCTCCGAAV GTGTCACGT). Transfection was conducted using Lipofectamine 2000 Reagent (Invitrogen, USA). 0.8 mg/mL of G418 was used for selecting stably transfected cells.

### Cell viability assays

Logarithmically growing cells were seeded in 96-well plates at a density of 2000 cells per well. Cell survival and proliferation were determined using the Cell Counting Kit 8 (CCK-8) (Dojindo, Japan). The medium was changed into a 100 μL fresh medium and a 10 μL CCK-8 solution for 1 h incubation when the OD value had to be measured. Varioskan Flash (Thermo Scientific, Finland) was used to detect OD values at 450 nm. The average of the 5 wells represented the result of each sample.

### Clonal efficiency assay

Cells were trypsinized into a single cell suspension and then cultured for 2 wk at a density of 200 per well. Colonies fixed with methanol were stained with crystal violet. Clones containing at least 50 cells were considered as one formation. Nonsense shRNA-transfected cells were used as controls.

### Statistical analysis

Statistical analyses were performed with SPSS ver. 23.0. *P <* 0.05 was considered statistically significant. Differences in the mRNA and protein expression levels of IDH1 between cancerous and paired paracancerous tissues were analyzed by paired Student's *t-test*. The Wilcoxon test was applied to analyze the difference in serum samples between patients with ESCC and healthy individuals. All samples were classified into high or low levels according to the cut-off value (the mean value of all participants). The Pearson correlation coefficient was determined to evaluate the relationship of the IDH1 expression in frozen tissue with that in serum. The correlations of the IDH1 in tissue or serum samples with the clinicopathological factors were determined using the bilateral *χ*^2^ test. The diagnostic value of serum IDH1 in ESCC was obtained using the ROC curve and the AUC. Survival curves were derived from the Kaplan–Meier method. The hazard ratios were assessed by univariate and multivariate Cox survival analyses. The AUC was also provided for the Cox regression models. For assays *in vitro*, the difference between each pair among sh-NC, sh-IDH2-1, and sh-IDH2-2 was evaluated using ANOVA with post-hoc Tukey test.
